# The Association Between Mental Well-Being and School Attendance Among Palestinian Adolescent Refugees in UNRWA Schools

**DOI:** 10.1007/s40653-022-00460-7

**Published:** 2022-05-28

**Authors:** Komal Nathani, Wei-Chen Lee, Shaden Taha, Masako Horino, Akihiro Seita, Hani Serag

**Affiliations:** 1 Department of Pediatrics, University of Texas Medical Branch (UTMB), Galveston, TX USA; 2 Department of Internal Medicine – Division of Endocrinology, University of Texas Medical Branch (UTMB), Galveston, TX, USA; 3 Department of Preventive Medicine and Population Health, University of Texas Medical Branch (UTMB), Galveston, TX USA; 4 Health Department, United Nations Relief and Works Agency for Palestine Refugees in the Near East (UNRWA), Amman, Jordan

**Keywords:** School attendance, Mental health, Gender, Child refugee

## Abstract

Adolescent refugees experience psychosocial stressors, including traumatic events, poverty, and loss of home and family. Exposure to conflict affects mental well-being in Palestinian adolescent refugees. Adolescent girls are among those vulnerable to post-traumatic stress associated with living in conflict zones, We assessed the association between reported mental well-being and school attendance among Palestinian adolescent refugees in UNRWA schools in Occupied Palestinian Territories, Jordan, Lebanon, and Syria. We also examined differences based on gender and place of residence, Palestinian adolescent refugees with certain mental well-being concerns were more likely to miss more days of school. Generally, females reported higher rates of loneliness and worry, but males were more likely to miss school. Gender-based differences were highest in Lebanon and least in the West Bank, More school-based and community-based mental well-being interventions are needed. Female-tailored programs are needed, especially in Palestinian refugee camps in Lebanon.

## Introduction

The health status of Palestinian refugees, including those living in the Occupied Palestinian Territories (OPT), is influenced by a vast array of complex factors. The consequences of the long-term occupation continue to affect people’s lives, livelihoods, and access to basic services, including health services. These consequences include military closures, restriction of people’s movement, forcible displacement, increased unemployment rates and poverty (especially in Gaza), land confiscation, a fragmented health system, and lack of coordination among key health services providers (Giacaman et al., 2009; Rosenthal, 2021). There are four major healthcare-providing organizations in the OPT: Palestinian Ministry of Health, established after the Oslo Accords in 1993; the United Nations Relief and Works Agency for Palestine Refugees in the Near East (UNRWA), founded in late 1949; nonprofit health organizations; and the private sector. About 5.5 million Palestinians are registered as refugees with UNRWA in Gaza, the West Bank, Lebanon, Syria, and Jordan.(Albanese & Takkenberg, 2020).

The health status of adolescents in the OPT, in particular, is a growing public health concern, given that nearly half of the population is under the age of 18 (UNICEF State of Palestine, 2018). Living under conditions of prolonged conflict has a negative impact on Palestinian adolescents (Giacaman et al., 2007). The child and adolescent refugee experience is characterized by a plethora of psychosocial stressors that can include traumatic events, poverty, and loss of home and family (Lustig et al., 2004). Previous research has found an association between exposure to conflict and rates of mental health disorders in Palestinian youth. These particular experiences have resulted in detrimental consequences on the social and emotional development of these children. Specifically, multiple exposures to violence and conflict in the OPT have been linked to higher rates of mental health and behavioral problems, including those related to depression, anxiety, and post-traumatic stress disorder (PTSD) (El-Khodary & Samara, 2020).

Gender-based differences in mental well-being among adolescent Palestinian refugees are under-studied. In their report on adolescent girls in Pettit et al. (2017) stated that adolescent girls are among the most vulnerable groups in terms of the impact of the endemic of post-traumatic stress associated with living in a conflict zone. They concluded that the mental well-being of adolescent females, more than that of adolescent males, is negatively affected by social isolation and the stigma of seeking mental health services (Pettit et al., 2017). Generally, epidemiological studies have shown that women are more likely to be affected by mood disorders such as anxiety and depression than men. This is seen worldwide, including in the Middle East (Seedat et al., 2009). The same is true for young Arab adolescents, with the burden of depressive symptoms and associated negative outcomes falling more heavily on girls than boys (Obermeyer et al., 2015). However, there is a lesser amount of broad epidemiological data studying the gender differences in mental health disorder manifestations between children and adolescents. Societal norms are likely at play regarding gender disparities in the care of mental health issues, such as the greater stigmatization of females as opposed to males accessing services (Ikkos, 2015). There has been an increased interest in developing gender-specific interventions in the Middle East to adress the the well-documented risk of poorer mental health outcomes for women and girls. However, so far there is only one article that is recently published on the potential of such measure in Qatar (a part of the region) (Alabdulla et al., 2022).

Existing literature on mental health in Arab societies postulates that care for mental well-being is most ideally delegated to the local community instead of hospitals. The institutionalization of mental health began in Palestine around 1922, when the British administration introduced a system of mental asylums (Giacaman et al., 2011). Western influences in addressing the psychological needs of the Palestinian population persisted throughout the following decades. In 2006, Palestinian health expenditure comprised only 2.5% for mental health, with the majority of this funding going towards psychiatric hospitals. Today, western approaches to addressing mental health problems are still emphasized by the existing sources of mental health services, despite a growing need for locally-led interventions focused on strengthening and healing the community (Giacaman et al., 2011).

Overall, mental health services for the Palestinian people are largely still in development, with some progress being made to transition away from institutionalization toward a more community-inclusive approach. Major challenges include underfunding of mental health resources and an overall lack of mental health professionals. Nonetheless, mental health services are provided through several different mediums throughout Palestine. In Gaza, non-governmental organizations (NGOs) play a critical role in delivering services, with UNRWA remaining the most prominent mental health service provider (The United Nations Relief and Works Agency for Palestine Refugees in the Near East UNRWA, 2016). UNRWA facilitates services through a community-oriented or school-based approach (Saymah et al., 2015). School-based mental health services is likely important resources for Palestinian youth.

Previous literature on mental health among refugees is limited, especially on studies of how suicide ideation and attempts may lead to negative outcomes, and how gender plays a role. This study examines the association between self-reported mental well-being in Palestinian adolescents and their school attendance in OPT (Gaza Strip and the West Bank), Jordan, Lebanon, and Syria. We also investigate gender differences in the association between mental well-being and school attendance. It also investigates gender differences in the association between mental well-being and school attendance.

## Methods

### Study Population

The Global School-Based Student Health Survey (GSHS) utilized a questionnaire developed primarily by the World Health Organization (WHO) to investigate the health behaviors and protective factors of school-aged individuals. In 2010, the GSHS was administered to Palestinian adolescents through distribution to schools in the Gaza Strip and the West Bank, as well as UNRWA Camps in the Gaza Strip, West Bank, Jordan, Lebanon, and Syria. A two-stage cluster design sample was used, in which schools were first randomly selected using a probability proportional to size approach. In the second stage, classes within the selected schools were randomly selected, and all students of the selected classes were allowed to participate in the survey. These self-administered questionnaires were anonymously filled out by students in 7th, 8th, and 9^th^ grade classrooms on a computer-scannable sheet.

### Measures

Our primary outcome was school attendance, assessed by the GSHS question, “*During the past 30 days, on how many days did you miss classes or school without permission*?” The answer choices included *0 days*, *1 or 2 days*, *3 to 5 days*, *6 to 9 days*, and *10 or more days*. Since our outcome responses are five different intervals, we performed an interval regression analysis and generated one variable for the lower point of the interval and another variable for the higher point of the interval. For instance, students who chose the third option "3 to 5 days" at least missed the school three days, and at maximum missed school five days. Instead of using the original variable, we created a new variable to indicate that the lowest point of missing days is equal to 3 and another variable to indicate that the highest point of missing days is equal to 5. We anticipate that this approach better captures the real value of each response than linear models.

To understand the gender and location-based differences in the association between mental well-being and school attendance among teenage students, we chose two stratifiers in this study. One was the student’s sex, where they answered either *male* or *female*. Another was the school’s location, including *Gaza, Jordan, Lebanon, Syria*, and the *West Bank*.

The exposures (independent variables) of the study were students’ reported feelings, suicide attempts, and relationships with other students at school. We assessed these exposures using the questions that asked students how often they felt lonely, worried about something, considered attempting suicide, made a suicide plan, and how many times they actually attempted suicide during the past 30 days. We also used the questions that asked how many close friends they have and how often most of the students in the school were kind and helpful towards them during the past 30 days.

### Analyses

Demographic characteristics were reported using frequency and proportions. Bivariate analyses with chi-squared tests were conducted to compare female students’ mental well-being and school attendance with that of male students. Given the gender differences in exposures and outcomes, we conducted moderation analysis to examine the hypothesis that gender moderates the association between students’ mental well-being and school attendance. After rejecting the null hypothesis, we conducted stratification analyses by gender and place of residence. While studying the association between mental well-being and school attendance for each gender, we further explored which mental well-being issue was more influential for students by gender.

All multivariate interval regression models were adjusted for students’ age because age is significantly associated with school attendance. To reflect its survey design, the default weight, stratum, and primary sampling unit (psu) variables were applied to all data analyses. Analyses were conducted using STATA v16, and a *p*-value smaller than 0.05 was considered significant.

## Study Ethics

The data we analyzed in this study were drawn from the WHO GSHS database. As a global survey, the study design and data collection protocols were reviews and approved by ethical review committees of the WHO and all countries in which the survey was implemented.

## Conceptual Framework

In Palestinian adolescents in UNRWA schools, we hypothesized that school attendance, measured by the number of days of missing classes, is influenced by mental well-being, measured by the feeling of loneliness, suicidal behavior, and peer relationships. However, this association is modifiable by gender and place of living.



## Results

### Demographic Characteristics

A total of 11,502 valid survey responses were collected, from students aged 11 to 16. After excluding respondents who did not report their sex or age (around 1.7% of the total sample) and applying weight variables, our final sample included 11,307 respondents representing the 184,334 total student population in this area (Table 1). Demographic characteristics of participants were revealed through survey questions about location, gender, and age. Areas included Gaza, Jordan, Lebanon, Syria, and the West Bank, with the most completed questionnaires from the Gaza Strip (33.5%) and Jordan (39.9%). The majority of participants (88.7%) were aged 13 to 15, with a mean age of 13.9. The sample was distributed approximately equally in terms of reported sex. Although the survey was voluntary, the response rate ranged was over 90% in all areas (World Health Organization (WHO), 2010).

**Table 1:** Demographics of Participants in The Global School-Based Student Health Survey (GSHS)

**Age**	Freq.	%
11	460	0.2%
12	13,974	7.6%
13	51,665	28.0%
14	60,623	32.9%
15	51,304	27.8%
16	6309	3.4%
mean	13.9	Std. = 0.05
**Sex**	Freq	%
Female	92,346	50.1%
Male	91,988	49.9%
**Area**	Freq.	%
Gaza	61,802	33.5%
Jordan	73,515	39.9%
Lebanon	11,187	6.1%
Syria	19,308	10.5%
West Bank	18,522	10.0%
Total	184,334	100.0%

### The Association Between Self-reported Mental Well-being and School Attendance

Of all the students who participated in the survey, 57.8% reported feelings of loneliness. Thirty-seven percent of students reported at least sometimes feeling a sense of worry that disturbed their ability to sleep at night. Nearly one-fifth (19.9%) of students reported suicidal ideation; among them, 17.2% reported having planned a suicide attempt. More than one-fifth (20.3%) of students reported at least one actual suicide attempt. With respect to self-perception of peer relationships, 80.6% of students had at least two close friends, and 73.8% of students reported that their peers were helpful and kind at least sometimes (Table 2).

**Table 2:** Distribution of Mental Well-Being and School of Attendance in Total and by Gender

	Freq	%	Freq	%	Freq	%
Feeling						
(1) Felt lonely*	Total (N = 182,439)		Male (n = 90,650)		Female (N = 91,7789)	
Never	77,059	42.2%	42,589	47.0%	34,470	37.6%
Rarely	46,362	25.4%	23,887	26.4%	22,475	24.5%
Sometime	30,889	16.9%	13,715	15.1%	17,174	18.7%
Most of the time	16,026	8.8%	6122	6.8%	9905	10.8%
Always	12,102	6.7%	4336	4.8%	7766	8.5%
(2) Worried about something*	Total (N = 181,617)		Male (N = 90,079)		Female (N = 91,538)	
Never	54,698	30.1%	32,531	36.1%	22,167	24.2%
Rarely	58,702	32.3%	29,599	32.9%	29,103	31.8%
Sometime	36,463	20.1%	16,565	18.4%	19,898	21.7%
Most of the time	21,578	11.9%	7642	8.5%	13,936	15.2%
Always	10,176	5.6%	3743	4.2%	6433	7.0%
Suicide Behavior						
**(1) Seriously consider attempting suicide**	Total (N = 180,298)		Male (N = 88,600)		Female (N = 91,698)	
Yes	35,929	19.9%	18,167	20.5%	17,762	19.4%
No	144,369	80.1%	70,433	79.5%	73,936	80.6%
**(2) Make a plan to attempt suicide**	Total (N = 179,139)		Male (N = 87,902)		Female (N = 91,237)	
Yes	30,778	17.2%	15,472	17.6%	15,306	16.8%
No	148,361	82.8%	72,430	82.4%	75,931	83.2%
(3) Number of times to attempt suicide*	Total (N = 181,877)		Male (N = 90,170)		Female (N = 91,707)	
0	145,041	79.7%	70,367	78.0%	74,674	81.4%
1	20,712	11.4%	10,578	11.7%	10,135	11.1%
2 or 3	9144	5.0%	4999	5.5%	4146	4.5%
4 or 5	3573	2.0%	2088	2.3%	1485	1.6%
6 or more	3407	1.9%	2138	2.4%	1268	1.4%
Peer Support						
(1) Number of close friends*	Total (N = 182,255)		Male (N = 90,644)		Female (N = 91,611)	
0	14,501	8.0%	7120	7.9%	7381	8.1%
1	20,899	11.5%	9509	10.5%	11,390	12.4%
2	24,952	13.7%	11,304	12.5%	13,648	14.9%
3 or more	121,903	66.9%	62,711	69.2%	59,192	64.6%
(2) Students in your school are kind*	Total (N = 179,955)		Male (N = 89,032)		Female (N = 90,923)	
Never	21,051	11.7%	14,873	16.7%	6178	6.8%
Rarely	26,063	14.5%	15,305	17.2%	10,758	11.8%
Sometimes	30,597	17.0%	16,676	18.7%	13,921	15.3%
Most of the time	36,244	20.1%	17,349	19.5%	18,895	20.8%
Always	66,000	36.7%	24,828	27.9%	41,171	45.3%
Outcome Measurement						
Number of days missing classes without permission*	Total (N = 180,626)		Male (N = 89,207)		Female (N = 91,418)	
0 days	112,931	62.5%	53,819	60.3%	59,112	64.7%
1 or 2 days	47,175	26.1%	22,672	25.4%	24,503	26.8%
3 to 5 days	12,142	6.7%	6985	7.8%	5157	5.6%
6 to 9 days	4143	2.3%	2584	2.9%	1559	1.7%
10 + days	4234	2.3%	3147	3.5%	1087	1.2%

Due to missing values, the total number of students varies in different questions

^*^Significant difference in the distribution between male and female students

### Gender and Location-based Variations

Our analysis revealed that more female students than male students *always* felt lonely (*p* < 0.001) and that more female students also reported *always* feeling worried (*p* < 0.001). There was no statistically significant difference between male and female students concerning suicidal ideation and planning. However, 2.4% of male students reported having attempted suicide *six or more times*, with 1.4% of female students attempting suicide the same number of times (Table 2). Regarding interpersonal relationships, more female students felt they did not have any close friends at school compared to boys who felt the same way. However, fewer male students felt that other students at school are kind.

Table 3 illustrates the association between mental well-being and school attendance stratified by gender and location. Among seven mental well-being questions, the number of attempted suicides was the best predictor of missing school. We found significant associations for both genders in the five locations, except for female students in Syria. This association was significant only in male students in Gaza, male students in Lebanon, and female students in the West Bank. Overall, we found that most of the seven mental well-being categories significantly determined male students’ school attendance, but not that of female students. Further, most mental well-being categories determined school attendance for male and female students in Gaza and Syria, but not the West Bank.

**Table 3:** The Association between Mental Well-Being and Days of Missing School for Both Genders and Five Regions

(Adjusted by Students' Age)		**Male Only (N = 91,988)**		**Female Only (N = 92,346)**	
		Coeff. (95%CI)	p-value	Coeff. (95%CI)	p-value
**Total**	Feel lonely	.245 (.167, .323)	< .001	.141 (.091, .190)	< .001
**(N = 1,843,34)**	Feel worried	.292 (.203, .381)	< .001	.099 (.049, .150)	< .001
	Consider suicide	.737 (.477, .998)	< .001	.509 (.268, .749)	< .001
	Plan suicide	.811 (.592, 1.029)	< .001	.397 (.218, .575)	< .001
	# of suicide attempts	.552 (.393, .710)	< .001	.344 (.160, .527)	< .001
	# of close friends	–.236 (–.450, –.023)	0.031	–.153 (–.258, –.049)	0.005
	Other students are kind	–.113 (–.159, –.067)	< .001	–.137 (–.181, –.093)	< .001
**Gaza**	Feel lonely	.279 (.174, .384)	< 0.001	.126 (.028, .224)	0.016
**(N = 61,802)**	Feel worried	.235 (.143, .327)	< 0.001	.099 (–.026, .225)	0.110
	Consider suicide	.763 (.369, 1.156)	0.001	.273 (.012, .535)	0.042
	Plan suicide	.811 (.565, 1.058)	< 0.001	.422 (.194, .649)	0.002
	# of suicide attempts	.606 (.375, .836)	< 0.001	.215 (–.049, .478)	0.101
	# of close friends	–.349 (–.621, –.078)	0.016	–.111 (–.258, .036)	0.125
	Other students are kind	–.114 (–.203, –.025)	0.016	-.111 (–.202, –.020)	0.020
**Jordan**	Feel lonely	.275 (.093, .457)	0.007	.161 (.061, .261)	0.004
**(N = 73,515)**	Feel worried	.344 (.123, .566)	0.006	.091 (–.008, .191)	0.068
	Consider suicide	.830 (.234, 1.426)	0.011	.769 (.238, 1.299)	0.008
	Plan suicide	.949 (.450, 1.447)	0.002	.398 (–.037, .833)	0.069
	# of suicide attempts	.501 (.153, .849)	0.009	.511 (.081, .942)	0.024
	# of close friends	–.164 (–.656, .328)	0.479	–.228 (–.475, .019)	0.067
	Other students are kind	–.086 (–.169, –.002)	0.045	–.151 (–.235, –.066)	0.002
**Lebanon**	Feel lonely	.257 (.096, .418)	0.003	.029 (–.011, .071)	0.145
**(N = 11,187)**	Feel worried	.408 (.278, .539)	< .001	.068 (–.003, .139)	0.058
	Consider suicide	.786 (.249, 1.323)	0.006	.320 (–.016, .657)	0.061
	Plan suicide	.666 (.146, 1.185)	0.015	.304 (–.061, .671)	0.097
	# of suicide attempts	.681 (.309, 1.053)	0.001	.281 (.045, .517)	0.022
	# of close friends	–.448 (–.649, –.247)	< .001	–.027 (–.092, .038)	0.391
	Other students are kind	–.228 (–.394, –.062)	0.010	–.017 (–.074, .039)	0.540
**Syria**	Feel lonely	.127 (.029, .225)	0.015	.136 (.082, .191)	< .001
**(N = 19,308)**	Feel worried	.158 (.062, .254)	0.003	.126 (.057, .196)	0.001
	Consider suicide	.654 (.307, 1.001)	0.001	.360 (–.031, .752)	0.069
	Plan suicide	.749 (.454, 1.044)	< .001	.307 (.061, .554)	0.017
	# of suicide attempts	.345 (.114, .575)	0.006	.196 (–.023, .415)	0.076
	# of close friends	–.098 –.277, .081)	0.247	–.046 (–.194, .102)	0.525
	Other students are kind	–.077 (–.124, –.029)	0.003	–.168 (–.272, –.065)	0.003
**West Bank**	Feel lonely	.087 (–.098, .272)	0.325	.068 (–.027, .163)	0.144
**(N = 18,522)**	Feel worried	.317 (.139, .496)	0.002	.058 (–.027, .143)	0.161
	Consider suicide	.196 (–.422, .815)	0.504	.268 (–.111, .647)	0.149
	Plan suicide	.259 (–.9607, 1.125)	0.527	.351 (.063, .639)	0.021
	# of suicide attempts	.647 (.331, .963)	0.001	.299 (.065, .534)	0.016
	# of close friends	–.093 (–.365, .180)	0.474	–.146 (–.275, –.018)	0.029
	Other students are kind	–.158 (–.345, .029)	0.090	–.094 (–.181, –.007)	0.037

**Fig. 1:**
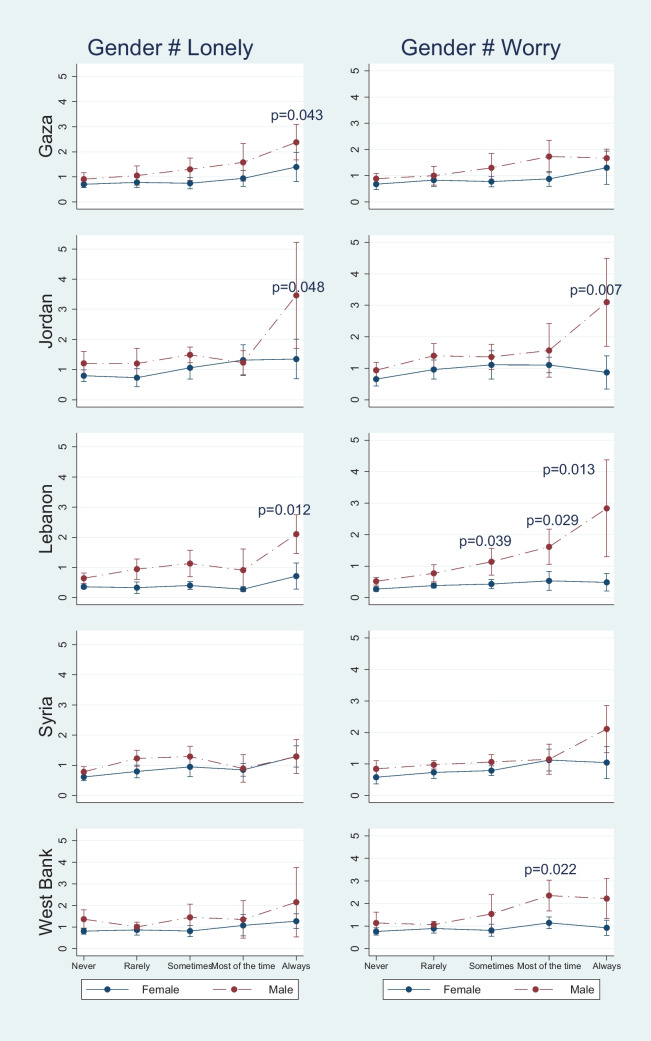
Compare Gender Differences in the Association between Feelings and School Attendance for Five Regions

**Fig. 2:**
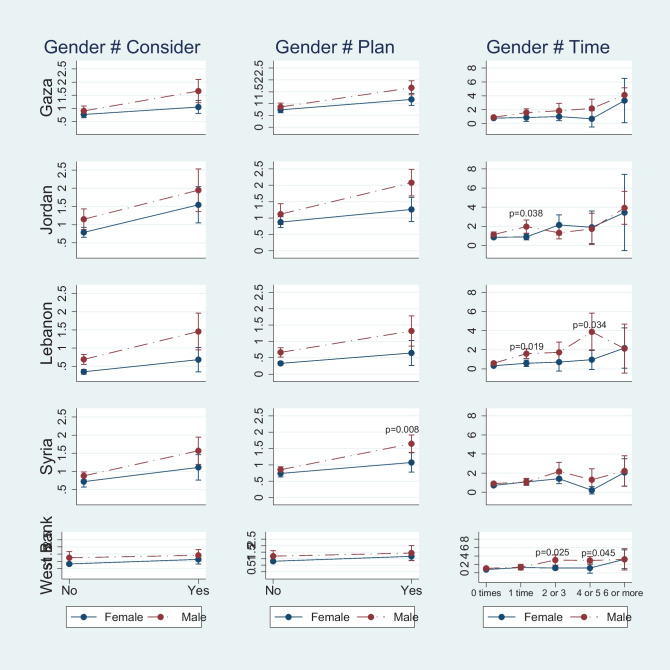
Compare Gender Differences in the Association between Suicide-Related Behaviors and School Attendance for Five Regions

**Fig. 3:**
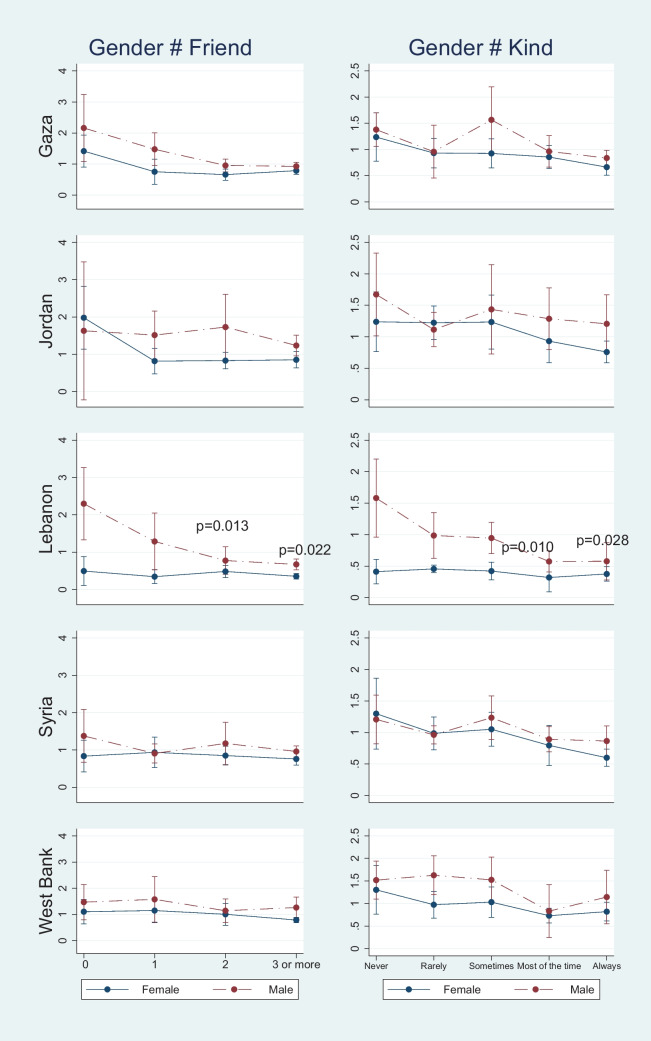
Compare Gender Differences in the Association between Peer Support and School Attendance for Five Regions

Figure 1 shows that male students in Gaza were more likely than female students to miss school if they always felt lonely (*p* < 0.05). Likewise, male students in Lebanon were more likely than female students to miss school if they were often worried about something or if they could not sleep at night. Figure 2 demonstrates that considering suicide did not determine school attendance for either gender. However, male students in Jordan (*p* = 0.038), Lebanon (*p* = 0.019), and the West Bank (*p* = 0.025) were more likely to miss school if they had attempted suicide at least once. Figure 3 illustrates that interpersonal relationships affect male students’ school attendance in Lebanon more than female students (*p* < 0.05). However, the gender difference was not significant in the other four areas.

## Discussion

The study showed a high prevalence of loneliness, worry, and suicidal ideation among adolescents in OPT (Gaza and West Bank) and Palestinian refugees in Lebanon, Syria, and Jordan. The analysis revealed that these psychological symptoms to be risk factors for decreased school attendance. It is important to recall that the mental health burden among Palestinians in these locations, in general, has been largely unmet and possibly exacerbated by the social, political, and economic challenges faced by this group. Limited availability of and access to mental health services is a significant barrier for Palestinians living in the OPT and those in UNRWA resettlement areas (Saymah et al., 2015). Given these realities, our study findings provide further evidence of unmet mental health needs in this population. We suggest more investments, in terms of financial and human resources, in mental health services by different healthcare-providing actors (The Palestinian Ministry of Health, UNRWA, healthcare-providing NGOs, and the private sector). We also suggest focusing on community mental health models rather than institution-based services. Comprehensive community mental health service delivery models will better reach out to those in need and ensure continuity of care while addressing the stigma associated with seeking mental health services (Flannery et al., 2011). These community models should serve as a primary care level with a robust referral system to institution-based higher levels of care, as needed.

This study indicated significant gender differences in the association between mental well-being and school attendance. Our analysis suggested that males were more likely to miss school in general, despite female students having higher rates of feeling lonely and worried. The analysis also showed differences in the frequency of suicidal attempts, six times higher in males than in females. Further studies are needed to identify and describe determinants of these gender-based differences. Gender inequalities in Palestinians in OPT and UNRWA served settlements in Jordan, Lebanon, and Syria are worsening in response to economic, social, and political demands. Further, this divide may have been exacerbated by recent COVID-19 lockdown measures (United Nations Office for the Coordination of Humaniterian Affairs OCHA, 2020). Traditional gender roles are common in Arab societies, where females are expected to marry, raise children, and take care of household duties, while males must provide financial stability and families often rely on sons to work from an early age. Punamaki et al. found that women reported lower lifetime trauma rates than men; however, their exposure to trauma was very similarly associated with PTSD. This result suggests that, although their experiences may be similar to those of males, females may be less likely to speak out, perhaps due to the social stigmas associated with gender inequalities in such patriarchal societies (Punamaki et al., 2005).

Palestinian youth are excluded from fully engaging in public and political life and struggle with pressures of gender roles defined by conservativism, patriarchy, and unbalanced social power. In addition to the Israeli military occupation, all these stresses adversely affect Palestinian adolescents’ well-being (The Institute of Community & Public Health - Birzeit University, 2021). Those who do not conform to traditional values, which include toleration of personal struggles for the sake of others, are often censored from their community (Fronk et al., 1999), adding to the stigma surrounding mental health in these regions. Nearly one-third of Palestinians are in need of mental health support, yet barriers such as social stigma, cultural views, religion, and lack of resources prevent them from receiving help (Marie et al., 2016; McKell & Hankir, 2018). Mental health research is also limited in Arab countries, which has restricted the development of adequate mental health services due to cultural beliefs and lack of resources. Of the 42 available mental health facilities, only 7% serve children, and even these are reserved for extreme neurotic cases (WHO and Ministry of Health of West Bank and Gaza, 2006).

In regard to schooling for Palestinian youth, over the last few decades, educational development has progressed through an increase in the number of schools and an increase in enrollment rates, especially among female students (UNICEF, The Situation of Palestinian Children in The Occupied Palestinian Territory, Jordan, Syria & Lebanon, 2011). Despite these advancements, the schooling of Palestinian youth is affected by several factors, including the restriction of movement, which presents a physical obstacle for some students attempting to go to school. This restriction also impedes the import of school supplies essential to the functioning of a classroom environment (UNICEF, The Situation of Palestinian Children in The Occupied Palestinian Territory, Jordan, Syria & Lebanon, 2011). Schooling is an integral part of human development. For Palestinian families, educational opportunity is perceived as one way for their children to secure social and economic security in the future despite ongoing displacement and oppression (Akesson, 2015). In addition, based on the findings of this study, we suggest that the school environment could serve as a point for Palestinian youth to access services to improve their mental well-being.

Further studies are warranted on youth mental well-being in Palestine. A comprehensive understanding of the psychological needs of this population can have a profound impact on the ability to create effective mental well-being interventions. The literature suggests that mental health is best addressed from a community-centered approach in Palestinian societies (Giacaman et al., 2011). For adolescents, school-based approaches represent a unique opportunity to both identify and address psychological symptoms. We suggest that such interventions have the advantage of being integrated into the school structure, allowing for universal availability of these services to all students and a gradual destigmatization of treatment. Public education can make way for mental health interventions for Palestinian adolescents. A previous study found that a school-based counseling program effectively alleviated psychological symptoms among Palestinian children and adolescents in Gaza. This study emphasized the advantages of school-based mental well-being programming that is versatile and accessible, offering much-needed interventions to this demographic (El-Khodary & Samara, 2019). Given that one-fifth of Palestinian adolescents reported attempting suicide at least once in our study, incorporating easily available school-based counseling may help identify and intervene in suicidal ideation in this young demographic.

Our analysis showed gender differences in psychological symptoms and school attendance. We therefore propose school-centered, gender-based studies and initiatives geared towards the mental health needs of Palestinian adolescents. The GSHS has been a valuable tool for assessing youth’s circumstances and health behaviors around the world. However, the discrepancy in male and female responses in the reported results of the GSHS of Palestinian adolescents calls for a more specific investigation into gender differences and their relation to manifested psychological stressors and outcomes.

## Limitations

Although this study consisted of a large sample of Palestinian students from several locations, including the Gaza Strip, the West Bank, Lebanon, Jordan, and Syria, only those from UNRWA schools were included, limiting the generalizability of the findings to adolescents in other areas. In addition, gender differences were a key focus of this study and gender role is greatly influenced by local cultures and social norms. The survey questions did not differentiate between sex and gender and consequently limited the ability of respondents to report their gender identity.

## Conclusion

This study illustrated an association between reported compromised mental well-being in Palestinian students and decreased school attendance. High rates of self-reported loneliness, worrying, and suicidality were noted in Palestinian youth, perhaps exacerbated by sociopolitical circumstances as well as the limited availability of resources for interventions. Psychological symptoms and school attendance differed between male and female students and by region, warranting a closer look into gender differences among Palestinian youth. There is a need for more comprehensive studies and the development of school-based and gender-based interventions in Palestinian adolescents to better understand and invest in their overall mental well-being.

### New Contribution to the Literature

This study responds to a knowledge gap in gender-based differences in mental well-being among adolescent Palestinian refugees. Accounting for these differences in this particular population and understanding their underlying causes will help in the design of effective culturally appropriate and gender-tailored interventions.
